# Neutrophil extracellular traps and phagocytosis in *Pythium insidiosum*

**DOI:** 10.1371/journal.pone.0280565

**Published:** 2023-01-24

**Authors:** Apichaya Sriwarom, Direkrit Chiewchengchol, Supichcha Saithong, Navaporn Worasilchai, Ariya Chindamporn

**Affiliations:** 1 Medical Microbiology, Interdisciplinary Program, Graduate School, Chulalongkorn University, Bangkok, Thailand; 2 Mycology Unit, Department of Microbiology, Faculty of Medicine, Chulalongkorn University, Bangkok, Thailand; 3 Center of Excellence in Translational Research in Inflammation and Immunology, Department of Microbiology, Faculty of Medicine, Chulalongkorn University, Bangkok, Thailand; 4 Immunology Unit, Department of Microbiology, Faculty of Medicine, Chulalongkorn University, Bangkok, Thailand; 5 Department of Transfusion Medicine and Clinical Microbiology, Faculty of Allied Health Science, Chulalongkorn University, Bangkok, Thailand; 6 Immunomodulation of Natural Products Research Group, Chulalongkorn University, Bangkok, Thailand; Ramathibodi Hospital, Mahidol University, THAILAND

## Abstract

Neutrophils are innate immune cells that play crucial roles in response to extracellular pathogens, including bacteria and fungi. *Pythium insidiosum* (*P insidiosum*) is a fungus-like pathogen that causes "pythiosis" in mammals. This study investigated *in vitro* function of human neutrophils against *P*. *insidiosum*. We demonstrated the killing mechanism of neutrophils when incubated with *P*. *insidiosum* zoospores (infective stage), such as phagocytosis and neutrophil extracellular traps (NETs). Healthy human neutrophils significantly reduced six strains of live zoospores isolated from different sources compared to the condition without neutrophils (*p* < 0.001), observed by colony count and trypan blue staining. As our results showed the killing ability of neutrophils, we further investigated the neutrophil killing mechanism when incubating with zoospores. Our study found that only two strains of heat-killed zoospores significantly induced phagocytosis (*p* < 0.01). Co-culture of heat-killed zoospores and neutrophils demonstrated NET formation, which was detected by immunofluorescence staining using DAPI, anti-myeloperoxidase, and anti-neutrophil elastase and quantitated under the fluorescence microscope. In addition, the level of cell-free DNA released from neutrophils (as a marker of NET production) after incubation with zoospores showed significantly increased levels when compared with unstimulated neutrophils (*p* < 0.001). Our findings demonstrate that neutrophils revealed the NET formation in response to *P*. *insidiosum* zoospores. This study is the first observation of the neutrophil mechanism against *P*. *insidiosum*, which could provide a better understanding of some parts of the innate immune response during pythiosis.

## Introduction

*Pythium insidiosum* (*P*. *insidiosum*) is a species of oomycetes fungus-like pathogen found in moist soil and swampy area [1, 2]. This pathogen causes "pythiosis" in mammals (e.g., humans, cattle, dogs, and cats), which usually occurs in tropical and subtropical areas [3–5]. The clinical manifestations of human infections can occur in four forms; vascular, cutaneous/subcutaneous, ocular, and disseminated pythiosis [6]. An asexual reproductive stage of this organism develops motile zoospores in a wet environment associated with its habitats and the prevalence area of this disease [7]. The infection starts once those zoospores attach to the host. After infection, the innate immune system is the primary line of defense to fight against invading pathogens, divided into two groups; barriers and protein molecules that prevent the infection and innate immune cells that recognize the pathogens and activate the effector cells [8]. However, understanding innate immune cells against *P*. *insidiosum* to reveal their pathogenesis has been limited. Thus, investigating neutrophils as a part of the innate immune response to *P*. *insidiosum* infection may provide a better insight into pathogenesis.

Neutrophils are important innate immune cells and are the most abundant polymorphonuclear leukocytes in human circulation, which play crucial roles during infection and inflammation [9, 10]. The main functions of neutrophils include phagocytosis, degranulation, reactive oxygen species (ROS) production, and neutrophil extracellular traps (NETs) formation [11, 12]. Evidence has shown that neutrophils are the first cells that migrate to the site of infection and eliminate pathogens [13, 14]. For fungal infection, the yeast form of *Candida albicans* (*C*. *albicans*) was phagocytosed by neutrophils, whereas pseudohyphae were entrapped by NETs [15]. Moreover, the hyphal form of *C*. *albicans* can induce ROS production generated by the NADPH oxidase system [16]. *Aspergillus fumigatus* (*A*. *fumigatus*) hyphae were killed by toxic granules released by neutrophils [17]. *Phialophora verrucosa* (*P*. *verrucosa*) conidia induced NET formation [18]. These data support that neutrophils play vital roles in response to several pathogens.

The immunological study of pythiosis based on the equine model showed that infectious zoospores and geminated hyphae activated host immune response, including T helper 2 (Th2) response, and white blood cell migration (e.g., eosinophil and basophil), which revealed the inflammation and tissue damage [19]. In contrast, infected animals who received *P*. *insidiosum* antigen (PIA) as immunotherapy demonstrated the Th1 response, stimulating cell-mediated cytotoxicity, which facilitated microbe killing [19, 20]. These findings were consistent with our previously unpublished data that showed increased levels of IFN-γ, IL-10, and IL-17 in Thai vascular pythiosis patients after receiving PIA immunotherapy. Noticeably, IL-17 activates neutrophil accumulation at the infection site [10, 21], suggesting that neutrophils are involved in the pathogenesis of pythiosis.

Only a few studies have demonstrated neutrophils’ role during pythiosis infection. This study investigated the functions of human neutrophils after co-culture with *P*. *insidiosum*, which are killing ability, phagocytosis, and NET formation.

## Materials and methods

### Blood collection and neutrophil isolation

Heparinized blood from healthy donors (n = 6) was collected by phlebotomy with written informed consent. This study was approved by the Institutional Review Board (IRB) of the Faculty of Medicine, Chulalongkorn University, Thailand (COA No: 572/2020). Fresh heparinized blood was layered on Polymorphprep^TM^ (Axis-shield, Norway) and centrifuged at 1,800 rpm for 30 min, 25°C for neutrophil isolation, according to the manufacturer’s instructions. The isolated neutrophils were collected, and cells were resuspended in RPMI 1640 medium with 25 mM HEPES and 2 mM L-glutamine (Gibco, USA), supplemented with 10% (v/v) fetal bovine serum (Gibco, USA). The contaminated red blood cells were lysed by 1x red cell lysis buffer (BioLegend, San Diego, CA). Cell viability and purity were determined by trypan blue and Wright-Giemsa staining, respectively.

### Microorganisms and zoospore production

Six strains of *P*. *insidiosum* from different sources were selected; ATCC 58643 (CBS 574.85) from an equine, CBS 101039 from an Indian patient, CBS 777.85 from an equine, PC10 from a Thai patient, ATCC 90586 from an American patient, and PEC1 from water environment in Pasak Chonlasith Dam, Thailand. These isolates were grown in Sabouraud dextrose broth (SDB, Oxoid, UK). The zoosporogenesis technique was modified from Mendoza and Prendas [22]. The pathogens were cultured on sterile grass leaves (*Axonopus compressus*). After incubating at 37˚C for 48 h, grass leaves with hyphae were further transferred into the induction medium to generate encysted zoospores. The induction medium contains calcium and magnesium ions that facilitate the development of zoospores by all *Pythium* species [5]. The encysted zoospores were harvested from the induction medium and washed with 1x phosphate-buffered saline (PBS, Biolegend, USA). Then, zoospore numbers were counted by a hemocytometer. All six strains of zoospores were individually used in all experiments; neutrophil killing assay, phagocytosis, and NET formation.

### Killing assay

The killing assay protocol was slightly modified from Gazendam RP *et al*. [17]. Co-culture of 2 × 10^5^ encysted zoospores opsonized with heat-inactivated pooled serum and 2 × 10^5^ neutrophils was performed for 2 h at 37°C in a 5% CO_2_ incubator. After incubation, neutrophils were lysed in sterile distilled water for 5 min. Samples were centrifuged at 10°C, 5,000 rpm for 10 min. Two assays observed the neutrophil-killing activity. The 0.4% trypan blue staining with a 1:4 ratio (zoospores:dye) was performed to show the nonviable zoospores under a 40x light microscope (Olympus BX50, Japan). Confirmation by colony count was done in addition to the staining. The samples were diluted with 1X PBS and then cultured on blood agar (Oxoid, UK), a recommended media for *P*. *insidiosum* culture [7]. After the incubation at 37°C for 15 h, colonies were counted and compared with the condition without neutrophils. All experiments were processed in triplicate independently.

### Phagocytosis

Neutrophil phagocytic activity was measured using pHrodo-succinimidyl ester (red fluorescence) (Invitrogen, USA). The staining protocols were slightly modified from Shintaku *et al*. [23]. Briefly, all zoospore isolates were heat-killed at 80°C for 30 min with a shaker, and the heat-killed zoospores were centrifuged for 10 min at 5,000 rpm. The pellets were resuspended in 663 μg/ml pHrodo in 1x PBS, incubated for 30 min with a shaker, and protected from light. Stained zoospores were washed three times with 1x PBS.

The pHrodo labeled zoospores were incubated with isolated neutrophils in a 96-U bottom well cell culture plate (SPL Life Sciences, Korea) with a ratio of 1:1 after opsonization with 5% (v/v) heat-inactivated pooled serum. The samples of each strain were treated with 10 μg/ml of cytochalasin D (Sigma-Aldrich, Israel) as a negative control because cytochalasin D inhibits actin polymerization and blocks cell movement resulting in phagocytosis inhibition [24]. After incubation for 30 min at 37°C in a 5% CO_2_ incubator, the samples were blocked with flow cytometry staining buffer (or FACS buffer) supplemented with human AB serum at 4°C for 20 min. The cells were stained with 0.2 mg/ml of anti-CD11b antibody (APC) as a neutrophil marker (Biolegend, USA) and washed with FACS buffer. Cells were stored on ice and then measured by a flow cytometer (FACsAria II, BD Biosciences, USA). Phagocytosed zoospores-pHrodo inside neutrophils were detected in the PE channel at 575 nm wavelength, which indicated PE-positive cells.

### NET formation

An immunofluorescence assay was used for NET formation from healthy human neutrophils induced with *P*. *insidiosum* zoospores. Sterile glass coverslips were placed in a 24-well flat-shaped bottom plate (Jet Bio-Filtration, China) and coated with poly-L-lysine (Sigma-Aldrich, Germany) for neutrophil attachment. After washing with 1xPBS, neutrophils were seeded and incubated for 1 h at 37°C in a 5% CO_2_ incubator. The heat-killed zoospores or 100 ng/ml phorbol myristate acetate (PMA, as a positive control, Sigma-Aldrich, USA) were added and incubated for 2 h. After incubation, the culture medium was gently aspirated for DNA quantification. The glass coverslips were fixed and permeabilized with 1% formaldehyde and 1X Tris-buffered saline (TBS) in 0.05% tween 20, respectively. The samples were blocked with 1xTBS with 2% bovine serum albumin (BSA) for 30 min. The staining protocol was slightly modified from Sae-khow *et al*. [25]. Samples were stained with 4’,6-Diamidino-2-phenylindole (DAPI, Thermo fisher scientific, USA), rabbit anti-neutrophil elastase, and mouse anti-myeloperoxidase (Abcam, UK) for DNA, elastase, and myeloperoxidase detection, respectively. Primary antibodies were detected with goat anti-rabbit IgG Alexa fluor 488 and goat anti-mouse IgG Alexa fluor 647 (Abcam, UK) under a fluorescence microscope (Olympus IX81, Japan) and confocal laser scanning microscope (ZEISS LSM800, Germany). The percentage of NET formation was calculated by counting the number of NET-forming cells per 100 neutrophils, which was processed in triplicate independently.

### Double-stranded DNA (dsDNA) quantification

After co-incubation for 2 h, the culture medium described in NET formation was prepared for dsDNA determination. 0.1 M CaCl_2_ and 50 U/ml micrococcal nuclease (Sigma-Aldrich, USA) were added for 10 min for DNA fragmentation and digestion. Next, 0.5 M ethylenediaminetetraacetic acid (EDTA) was added to stop the reaction. Then, the supernatants were collected and incubated with Quant-iT^TM^ PicoGreen® reagent (Invitrogen, UK) in a ratio of 1:1 to determine the cell-free DNA, according to the manufacturer’s instructions. After incubation for 5 min in the dark, the DNA mixture was measured at an excitation wavelength of 480 nm and emission wavelength of 530 nm by spectrofluorometer (Varioskan Flash, Thermo fisher scientific, Finland).

### Statistical analyses

Statistical analyses were performed by GraphPad version 5.03 (GraphPad Software, San Diego, CA). Data are presented as mean ± standard error of the mean (SEM). One-way ANOVA followed by Bonferroni analysis was used to compare the groups. Differences with a p-value of * *p* < 0.05, ** *p* < 0.01, and *p* < 0.001 were considered statistically significant.

## Results

### Inhibition of *P*. *insidiosum* growth and viability by human neutrophils

**Fig 1:**
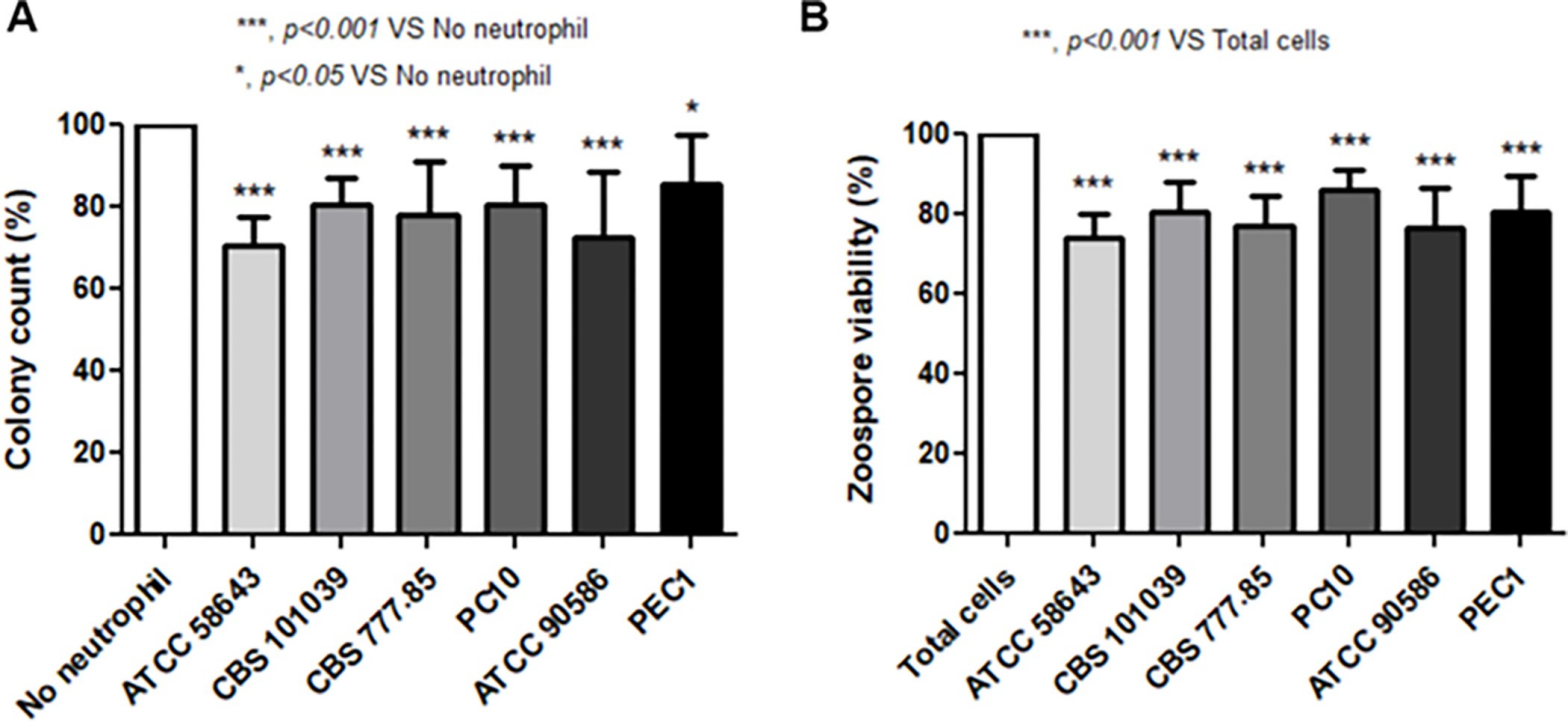
The killing activity of neutrophils against different strains of *P*. *insidiosum* zoospores. (A) Neutrophils from healthy donors (n = 6) were incubated with opsonized zoospores for 2 h. The percentage of colony counts on blood agar from each strain was calculated and compared with the same strain without neutrophils (100%). (B) The viability of zoospores after co-culture with or without neutrophils was observed using trypan blue staining under a light microscope, 400x magnification. The percentage of zoospore viability was calculated and compared with the same strain without neutrophils (100%). (*** *p* < 0.001, * *p* < 0.05).

The killing activity of healthy neutrophils against six strains of *P*. *insidiosum* zoospores was evaluated. The results demonstrated that the percentages of zoospore viability were significantly reduced in all strains of *P*. *insidiosum* after co-culture with neutrophils; ATCC 58643 (74.0 ± 6.1%), ATCC 90586 (76.6 ± 10.2%), CBS 777.85 (77.1 ± 7.7%), CBS 101039 (80.3 ± 7.8%), PEC1 (80.4 ± 9.3%), and PC10 (86.1 ± 5.2%) (*p* < 0.001, n = 6). Moreover, the percentages of colony count on blood agar were significantly decreased in all strains of *P*. *insidiosum* after co-culture with neutrophils; ATCC 58643 (70.7 ± 6.7%), ATCC 90586 (72.4 ± 16.3%), CBS 777.85 (78.2 ± 13.1%), CBS 101039 (80.4 ± 6.4%), PC10 (80.6 ± 9.7%) (*p* < 0.001, n = 6), and PEC1 (85.8 ± 11.7%) (*p* < 0.05, n = 6), respectively when compared with zoospores without neutrophils (100%) (Fig 1).

### *P*. *insidiosum* phagocytosed by human neutrophils

**Fig 2:**
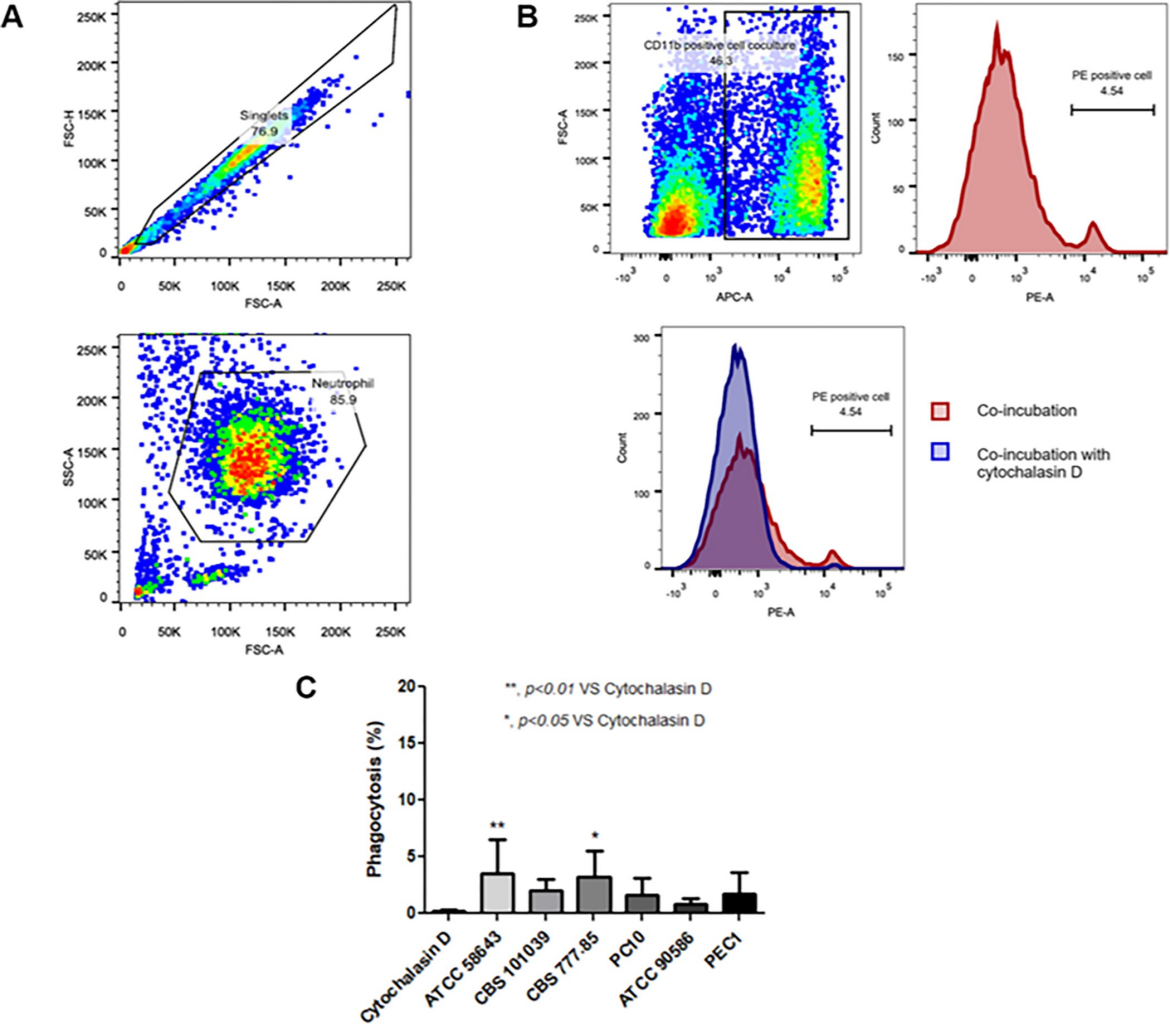
Phagocytosed zoospores from six strains of *P*. *insidiosum* by human neutrophils (n = 6). (A) Representative flow cytometry result of gated neutrophils after co-culture with zoospores. (B) Representative histogram pictures of gated neutrophils and PE-positive cells indicating phagocytosed zoospores of *P*. *insidiosum* and gated neutrophils pretreated with or without cytochalasin D (negative control). (C) The percentages of phagocytosis after neutrophils incubated with zoospores compared with cytochalasin D treatment in each strain. (** *p* < 0.01, * *p* < 0.05).

As neutrophils reduced colony counts and zoospore viability of *P*. *insidiosum*, we investigated whether neutrophils eliminate this pathogen through phagocytosis. Healthy neutrophils were incubated with opsonized heat-killed pHrodo labeled *P*. *insidiosum* zoospores. Phagocytosed zoospores-PE inside neutrophils (PE-positive cells) were gated and detected using flow cytometry. Neutrophils pretreated with cytochalasin D were used as a negative control (Fig 2A and 2B). The results showed that four strains of zoospores, PEC1, CBS 777.85, CBS101039, and ATCC 58643, were phagocytosed by neutrophils. However, the percentages of phagocytosis were significantly increased only in zoospores from ATCC 58643 and CBS 777.85 strains; 3.6 ± 1.2% (*p* < 0.01) and 3.2 ± 0.9% (*p* < 0.05, n = 6), respectively when compared with the negative control (Fig 2C).

### Induction of NET formation by *P*. *insidiosum*

**Fig 3:**
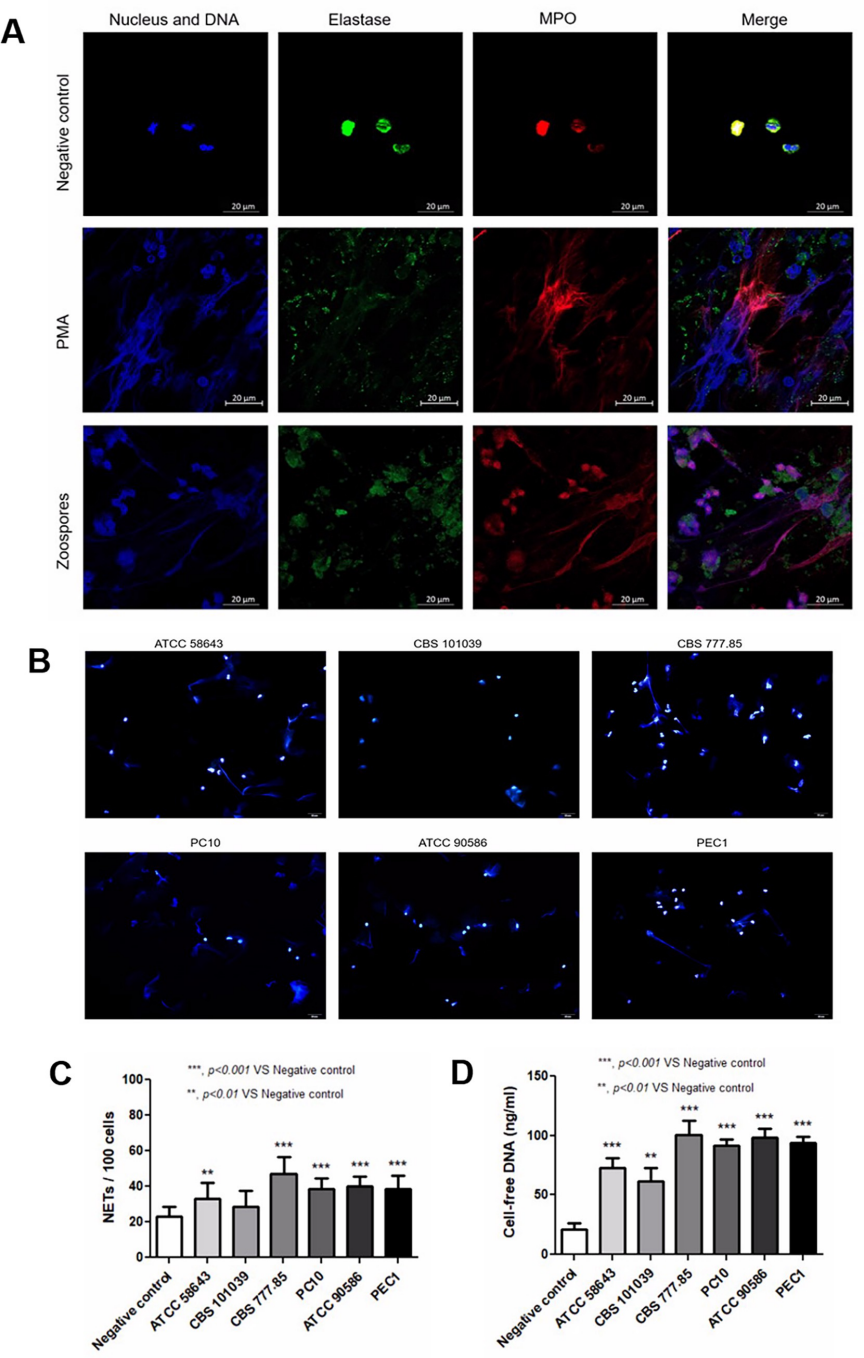
The NET counts and the levels of cell-free DNA when human neutrophils (n = 6) were treated with zoospores from six strains of *P*. *insidiosum*. (A) Representative fluorescence images captured by confocal microscopy (original magnification 630X and scale bars indicated object size as labeled 20 μm) of neutrophils co-cultured with PMA (100 ng/ml, as a positive control) or zoospores from CBS 777.85 strain, stained with DAPI (blue), neutrophil elastase (green) and myeloperoxidase (red), and merged images for NET identification. (B) Representative pictures of NETs released by neutrophils per 100 cells under a fluorescence microscope (n = 6 for all groups), stained with DAPI (blue), 400x magnification. (C) The percentages of NET-released neutrophils compared with negative control (unstimulated neutrophils) counted NETs per 100 neutrophils. (D) The levels of cell-free DNA in supernatants from neutrophils co-cultured with zoospores. (*** *p* < 0.001, ** *p* < 0.01).

We further investigated whether zoospores of *P*. *insidiosum* induced NET formation. Our preliminary results showed that zoospores from CBS 777.85 strain induced NET released from human neutrophils (Fig 3A). After incubation, cells were stained for the nucleus and released DNA (blue), elastase (green), and MPO (red), which demonstrated blue web-like structures containing green elastase and red MPO granules. NET counts were significantly increased when neutrophils were treated with zoospores from all six strains of *P*. *insidiosum*; CBS 777.85 (48 ± 9.3%), ATCC 90586 (41 ± 5.6%), PEC1 (39 ± 7.5%), PC10 (39 ± 6.3%) (*p* < 0.001, n = 6), ATCC 58643 (33 ± 9.3%) (*p* < 0.01, n = 6), when compared with untreated neutrophils (Fig 3B and 3C). However, the levels of cell-free DNA (as a marker of NET products in cell culture medium) were significantly increased when neutrophils were treated with zoospores from all strains of *P*. *insidiosum*; CBS 777.85 (100.80 ± 12.17 ng/ml), ATCC 90586 (98.73 ± 7.08 ng/ml), PEC1 (93.68 ± 5.40 ng/ml), PC10 (91.65 ± 4.92 ng/ml), ATCC 58643 (72.70 ± 8.42 ng/ml) (*p* < 0.001, n = 6), and CBS 101039 (61.53 ± 11.01 ng/ml) (*p* < 0.01, n = 6), when compared with untreated neutrophils (Fig 3D).

## Discussion

Pythiosis is a life-threatening disease caused by *P*. *insidiosum*, the fungus-like microbe [1, 4, 7]. In the initial phase of infection, the innate immune system acts as the primary line of defense to fight against invading pathogens [8]. Understanding the human immune response to *P*. *insidiosum*, especially innate immunity, is still unclear [26–28]. Neutrophils are one of the important granulocytic innate immune cells that play essential roles in response against pathogens [13]. However, no report demonstrates neutrophils’ response against *P*. *insidiosum*. This study, therefore, investigated the killing ability of neutrophils and two primary neutrophil mechanisms, which were composed of phagocytosis and NET formation, in response to *P*. *insidiosum*.

In this study, we first explored the killing activity of human neutrophils against *P*. *insidiosum*. We determined whether human neutrophils decreased the colony number and viability of zoospores (infective stage) of *P*. *insidiosum*. As predicted, all different strain colony counts on blood agar were significantly reduced when neutrophils were added to the zoospores. The significant reduction of zoospore viability supported this result after these zoospores were co-cultured with human neutrophils and stained with trypan blue to discriminate live/dead zoospores [29]. However, complete colony count reductions and zoospore viability were not observed. These findings indicate that neutrophils are one part that plays a role in the elimination of *P*. *insidiosum*. Nevertheless, our results suggest that *P*. *insidiosum* was perhaps hardly destroyed by human neutrophils.

Phagocytosis is one of the significant mechanisms of neutrophils in response to invading pathogens. This study, therefore, investigated the phagocytosis of neutrophils in *P*. *insidiosum*. There were only 2 strains that showed significantly increased neutrophil phagocytosis. These findings suggest that phagocytosis is probably not the primary killing mechanism of *P*. *insidiosum* because the size of the zoospores of this pathogen is large, and neutrophils are probably unable to phagocytose zoospores of this pathogen easily. In addition, human neutrophils are 10–12 μm in diameter, while zoospores of *P*. *insidiosum* are 8–12 μm in diameter and are larger than the yeast form of *C*. *albicans* (2–3 μm in diameter) [30, 31]. Branzk N *et al*. reported that the incubation of neutrophils and yeast-locked *C*. *albicans* induced neutrophil phagocytosis. In contrast, the incubation with the hyphal form of this *C*. *albicans* resulted in NET formation [15]. These data supported that phagocytosis of neutrophils depended on the microbial size and implied that neutrophils formed NETs when they encountered large-sized pathogens such as *P*. *insidiosum*.

As NETs are another essential mechanism of neutrophils in fungal infection, particularly large-sized pathogens (e.g., zoospores of *P*. *insidiosum*), this study determined NET formation after human neutrophils were incubated with *P*. *insidiosum* zoospores. Neutrophils released the DNA web-like structure containing elastase and MPO granules but were not observed in untreated neutrophils. In addition, the levels of NET products or cell-free DNA supported these findings. Neutrophils demonstrate the essential roles of NET in response to many fungi, such as *Pialophora verrucosa* [18], *Aspergillus fumigatus* [17], *Curvularia lunata* [32], etc., resulting in the production of NADPH oxidase, MPO and elastase releasing, and decreasing of fungal viability. Our observation supports that NET formation may be the primary killing mechanism of *P*. *insidiosum*.

In conclusion, this study demonstrated that human neutrophils kill *P*. *insidiosum* via NET formation and partly phagocytosis. As large-sized pathogens such as *P*. *insidiosum* zoospores are the infective stage causing pythiosis, this study supported the idea that NETs become a key player in eliminating these pathogens. However, these observations could not represent the exact pathogenesis of neutrophils during *P*. *insidiosum* infection because heat-killed zoospores were used in both phagocytosis and NET assays. Dead microorganisms show lower immune stimulation than live pathogenic microorganisms due to their virulence factors and some protein molecules [33]. Killed zoospores lack some activities (e.g., hyphal germination) and some secretory components (e.g., protease), which may decrease the ability of immune stimulation. Confirming phagocytosis and NET formation by human neutrophils incubating with live zoospores is needed. Moreover, future studies should increase the neutrophil population in the case of healthy donors and pythiosis patients and zoospores induced from different hosts to explore how neutrophils function. This information will shed light on new management and therapy in pythiosis patients.

## References

[1] Austwick P, Copland J. Swamp cancer. Nature. 1974;250(5461):84–. doi: 10.1038/250084a0 4276247

[2] Lévesque CA. Fifty years of oomycetes—from consolidation to evolutionary and genomic exploration. Fungal Diversity. 2011;50(1):35–46.

[3] Konradt G, Bassuino DM, Bianchi MV, Castro L, Caprioli RA, Pavarini SP, et al. Cutaneous pythiosis in calves: An epidemiologic, pathologic, serologic and molecular characterization. Medical mycology case reports. 2016;14:24–6. doi: 10.1016/j.mmcr.2016.11.004 28050341 PMC5192245

[4] Mendoza L, Vilela R. The mammalian pathogenic fungal-like oomycetes. Mycoses. 2012;55:32–3.

[5] Gaastra W, Lipman LJ, De Cock AW, Exel TK, Pegge RB, Scheurwater J, et al. Pythium insidiosum: an overview. Veterinary microbiology. 2010;146(1–2):1–16. doi: 10.1016/j.vetmic.2010.07.019 20800978

[6] Krajaejun T, Sathapatayavongs B, Pracharktam R, Nitiyanant P, Leelachaikul P, Wanachiwanawin W, et al. Clinical and epidemiological analyses of human pythiosis in Thailand. Clinical Infectious Diseases. 2006;43(5):569–76. doi: 10.1086/506353 16886148

[7] Permpalung N, Worasilchai N, Chindamporn A. Human Pythiosis: Emergence of Fungal-Like Organism. Mycopathologia. 2019:1–12. 31845178 10.1007/s11046-019-00412-0

[8] Alberts B, Johnson A, Lewis J, Raff M, Roberts K, Walter P. Innate immunity. Molecular Biology of the Cell 4th edition: Garland Science; 2002.

[9] Firestein GS, Budd RC, Gabriel SE, McInnes IB, O’Dell JR. Kelley and Firestein’s textbook of rheumatology: Elsevier Health Sciences; 2016.

[10] Kolaczkowska E, Kubes P. Neutrophil recruitment and function in health and inflammation. Nature reviews immunology. 2013;13(3):159–75. doi: 10.1038/nri3399 23435331

[11] Neutrophils Borregaard N., from marrow to microbes. Immunity. 2010;33(5):657–70. 21094463 10.1016/j.immuni.2010.11.011

[12] Amulic B, Cazalet C, Hayes GL, Metzler KD, Zychlinsky A. Neutrophil function: from mechanisms to disease. Annual review of immunology. 2012;30:459–89. doi: 10.1146/annurev-immunol-020711-074942 22224774

[13] Rosales C. Neutrophil: a cell with many roles in inflammation or several cell types? Frontiers in physiology. 2018;9:113. doi: 10.3389/fphys.2018.00113 29515456 PMC5826082

[14] Nauseef WM, Borregaard N. Neutrophils at work. Nature immunology. 2014;15(7):602–11. doi: 10.1038/ni.2921 24940954

[15] Branzk N, Lubojemska A, Hardison SE, Wang Q, Gutierrez MG, Brown GD, et al. Neutrophils sense microbe size and selectively release neutrophil extracellular traps in response to large pathogens. Nature immunology. 2014;15(11):1017–25. doi: 10.1038/ni.2987 25217981 PMC4236687

[16] Gazendam RP, van Hamme JL, Tool AT, van Houdt M, Verkuijlen PJ, Herbst M, et al. Two independent killing mechanisms of Candida albicans by human neutrophils: evidence from innate immunity defects. Blood, The Journal of the American Society of Hematology. 2014;124(4):590–7. doi: 10.1182/blood-2014-01-551473 24948657

[17] Gazendam RP, van Hamme JL, Tool AT, Hoogenboezem M, van den Berg JM, Prins JM, et al. Human neutrophils use different mechanisms to kill Aspergillus fumigatus conidia and hyphae: evidence from phagocyte defects. The Journal of Immunology. 2016;196(3):1272–83. doi: 10.4049/jimmunol.1501811 26718340

[18] Liu Q, Yi W, Jiang S, Song J, Liang P. Neutrophil Extracellular Traps Serve as Key Effector Molecules in the Protection Against Phialophora verrucosa. Mycopathologia. 2021;186(3):367–75. doi: 10.1007/s11046-021-00554-0 34013384 PMC8249254

[19] Mendoza L, Newton JC. Immunology and immunotherapy of the infections caused by Pythium insidiosum. Medical Mycology. 2005;43(6):477–86. doi: 10.1080/13693780500279882 16320491

[20] Yolanda H, Krajaejun T. History and perspective of immunotherapy for pythiosis. Vaccines. 2021;9(10):1080. doi: 10.3390/vaccines9101080 34696188 PMC8539095

[21] Hernández-Santos N, Gaffen SL. Th17 cells in immunity to Candida albicans. Cell host & microbe. 2012;11(5):425–35. 22607796 10.1016/j.chom.2012.04.008 PMC3358697

[22] Mendoza L, Prendas J. A method to obtain rapid zoosporogenesis of Pythium insidiosum. Mycopathologia. 1988;104(1):59–62. doi: 10.1007/BF00437925 3216883

[23] Shintaku T, Glass KA, Hirakawa MP, Longley SJ, Bennett RJ, Bliss JM, et al. Human endothelial cells internalize Candida parapsilosis via N-WASP-mediated endocytosis. Infection and immunity. 2013;81(8):2777–87. doi: 10.1128/IAI.00535-13 23690407 PMC3719575

[24] Goodman SR. Medical cell biology: Academic Press; 2007.

[25] Sae-Khow K, Tachaboon S, Wright HL, Edwards SW, Srisawat N, Leelahavanichkul A, et al. Defective neutrophil function in patients with sepsis is mostly restored by ex vivo ascorbate incubation. Journal of Inflammation Research. 2020;13:263. doi: 10.2147/JIR.S252433 32636666 PMC7326689

[26] Kirzhner M, Arnold SR, Lyle C, Mendoza LL, Fleming JC. Pythium insidiosum: a rare necrotizing orbital and facial infection. Journal of the Pediatric Infectious Diseases Society. 2015;4(1):e10–e3. doi: 10.1093/jpids/piu015 26407370

[27] Wongprompitak P, Pleewan N, Tantibhedhyangkul W, Chaiprasert A, Prabhasawat P, Inthasin N, et al. Involvement of Toll-like receptor 2 on human corneal epithelium during an infection of Pythium insidiosum. Asian Pacific Journal of Allergy and Immunology. 2020;38(2):129–38. doi: 10.12932/AP-110518-0311 30118247

[28] Ledur PC, Tondolo JS, Jesus FP, Verdi CM, Loreto ÉS, Alves SH, et al. Dendritic cells pulsed with Pythium insidiosum (1, 3)(1, 6)-β-glucan, Heat-inactivated zoospores and immunotherapy prime naïve T cells to Th1 differentiation in vitro. Immunobiology. 2018;223(3):294–9. 29074300 10.1016/j.imbio.2017.10.033

[29] Fang I-J, Trewyn BG. Application of mesoporous silica nanoparticles in intracellular delivery of molecules and proteins. Methods in Enzymology. 2012;508:41–59. doi: 10.1016/B978-0-12-391860-4.00003-3 22449920

[30] Mukaremera L, Lee KK, Mora-Montes HM, Gow NA. Candida albicans yeast, pseudohyphal, and hyphal morphogenesis differentially affects immune recognition. Frontiers in immunology. 2017;8:629. doi: 10.3389/fimmu.2017.00629 28638380 PMC5461353

[31] Erwig LP, Gow NA. Interactions of fungal pathogens with phagocytes. Nature Reviews Microbiology. 2016;14(3):163–76. doi: 10.1038/nrmicro.2015.21 26853116

[32] Tóth EJ, Varga M, Takó M, Homa M, Jáger O, Hermesz E, et al. Response of human neutrophil granulocytes to the hyphae of the emerging fungal pathogen Curvularia lunata. Pathogens. 2020;9(3):235. doi: 10.3390/pathogens9030235 32245253 PMC7157731

[33] Pearlman E, Sun Y, Roy S, Karmakar M, Hise AG, Szczotka-Flynn L, et al. Host defense at the ocular surface. International reviews of immunology. 2013;32(1):4–18. doi: 10.3109/08830185.2012.749400 23360155 PMC3750950

